# Effect of Testosterone on Insulin Stimulated IRS1 Ser Phosphorylation in Primary Rat Myotubes—A Potential Model for PCOS-Related Insulin Resistance

**DOI:** 10.1371/journal.pone.0004274

**Published:** 2009-01-26

**Authors:** Michael C. Allemand, Brian A. Irving, Yan W. Asmann, Katherine A. Klaus, Laura Tatpati, Charles C. Coddington, K. Sreekumaran Nair

**Affiliations:** 1 Department of Obstetrics and Gynecology, Division of Reproductive Endocrinology and Infertility, Mayo Clinic Graduate School of Medicine, Rochester, Minnesota, United States of America; 2 Department of Internal Medicine, Endocrine Research Unit, Mayo Clinic Graduate School of Medicine, Rochester, Minnesota, United States of America; National Institute of Child Health and Human Development/National Institutes of Health, United States of America

## Abstract

**Background:**

Polycystic ovary syndrome (PCOS) is characterized by a hyperandrogenic state and frequently develops skeletal muscle insulin resistance. We determined whether testosterone adversely affects insulin action by increasing serine phosphorylation of IRS-1^636/639^ in differentiated rat skeletal muscle myotubes. The phosphorylation of Akt, mTOR, and S6K, downstream targets of the PI3-kinase-IRS-1 complex were also studied.

**Methods:**

Primary differentiated rat skeletal muscle myotubes were subjected to insulin for 30 min after 16-hour pre-exposure to either low (20 ng/ml) or high (200 ng/ml) doses of testosterone. Protein phosphorylation of IRS-1 Ser^636/639^, Akt Ser^473^, mTOR-Ser^2448^, and S6K-Thr^389^ were measured by Western blot with signal intensity measured by immunofluorescence.

**Results:**

Cells exposed to 100 nM of insulin had increased IRS-1 Ser^636/639^ and Akt Ser^473^ phosphorylation. Cells pre-exposed to low-dose testosterone had significantly increased insulin-induced mTOR-Ser^2448^ and S6K-Thr^389^ phosphorylation (*p*<0.05), and further increased insulin-induced IRS-1 Ser^636/639^ phosphorylation (*p* = 0.042) compared to control cells. High-dose testosterone pre-exposure attenuated the insulin-induced mTOR-Ser^2448^ and S6K-Thr^389^ phosphorylation.

**Conclusions:**

The data demonstrated an interaction between testosterone and insulin on phosphorylation of intracellular signaling proteins, and suggests a link between a hyperandrogenic, hyperinsulinemic environment and the development of insulin resistance involving serine phosphorylation of IRS-1 Ser^636/639^. These results may guide further investigations of potential mechanisms of PCOS-related insulin resistance.

## Introduction

Polycystic Ovary Syndrome (PCOS) is estimated to affect approximately 5–7% of premenopausal women in the U.S., and is associated with a significant risk of developing type 2 diabetes (T2D) independent of obesity [1]. PCOS is characterized by a hyperandrogenic state and exposure to exogenous testosterone (T) *in vivo* has been associated with insulin resistance in rats and human females [2]–[4]. However, the mechanism by which hyperandrogenism results in the development of insulin resistance remains incompletely defined. The risk of developing the metabolic syndrome in adolescent females with PCOS correlates strongly with increased bioavailable T concentrations, independent of obesity [5]. In addition, a recent systematic review demonstrated a significantly higher risk of developing T2D mellitus in women with elevated T concentrations [6].

Insulin receptor substrate-1 (IRS-1) is a signaling protein which couples the insulin receptor to the phosphoinositide-3-kinase (PI3K) signaling cascade [7]. Serine phosphorylation of IRS-1 disassociates coupling of IRS-1 signal transduction to PI3K and results in insulin resistance. Exposure to a number of metabolites, including free fatty acids, glucose, diacylglycerol, and fatty acyl-CoA's increase serine phosphorylation of IRS-1 and result in impaired insulin signaling [8]. Skeletal muscle and liver cells from obese rats have been shown to demonstrate elevated IRS-1 serine phosphorylation and impaired insulin signaling through the PI3K pathway [9]. In addition, IRS-1 associated PI3K activity has been shown to be decreased *in vivo* in skeletal muscle of PCOS patients, and cultured skeletal muscle from PCOS patients has been shown to have elevated levels of IRS-1 serine phosphorylation as compared to age and body mass index (BMI) matched controls [10], [11].

The purpose of the current study was to test the hypothesis that T exposure increases serine phosphorylation of IRS-1 in skeletal muscle, the dominant site of insulin mediated glucose uptake in the postprandial state, in a hyperinsulinemic environment. Previous studies have shown that increased phosphorylation of mTOR and S6K causes sub-cellular redistribution of IRS-1, and inactivates IRS-1 by increasing its serine phosphorylation leading to insulin resistance [9], [12]–[15].

In an effort to further investigate aberrations in insulin signaling under these experimental conditions, we also studied the phosphorylation of Akt, mTOR, and S6K, which lie downstream of the PI3-kinase-IRS-1 complex.

## Results

### Homogeneity of myoblast culture

**Figure 1:**
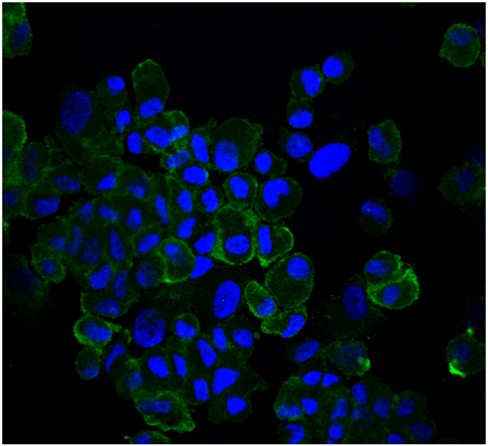
There was no contamination of non-myoblast cells in our primary culture of rat skeletal muscle cells. Cells were positively stained with MHCI-antibody (Green Florescence).

Immunohistochemistry staining of primary cultured rat myoblast cells showed positive MHC-1 in >95% of the cells, confirming the successful isolation and primary culture of skeletal muscle cells (Figure 1).

### The impact of T exposure on the phosphorylation of insulin signaling proteins

#### IRS-1

**Figure 2:**
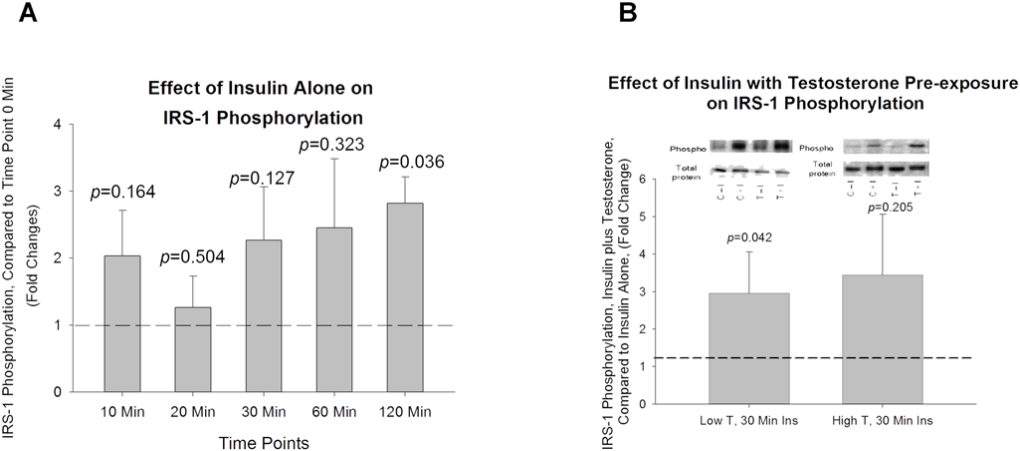
The effect of insulin in IRS-1 phosphorylation with or without pre-exposure of low and high dose testosterone. (A). The effect of insulin alone on IRS-1 phosphorylation after insulin treatment for 10, 20, 30, 60, and 120 minutes; Insulin significantly increased IRS-1 Ser^636/639^ phosphorylation after 120 minutes; (B). Cells were pre-exposed to low-dose (20ng/ml) or high-dose (200ng/ml) of testosterone for 16 hours before insulin treatment. Compared to non-testosterone treated (control) cells, IRS-1 Ser^636/639^ phosphorylation was significantly increased with both low and high-dose testosterone exposure. Representative western blots are shown above the bar graphs. The four experimental groups are: control cells without insulin (C−I), control cells plus insulin (C+I), testosterone exposed cells without insulin (T−I), and testosterone exposed cells plus insulin (T+I). The phosphorylated signal of IRS-1 Ser^636/639^ is normalized to total IRS-1 in the sample.

Exposure of differentiated rat skeletal muscle myotubes to 100 nM insulin significantly increased phosphorylation of IRS-1 Ser^636/639^ at 120 Min (*p* = 0.036,Figure 2A). Phosphorylation of IRS-1 Ser^636/639^ was also elevated at 30 and 60 Min (Figure 2A), however, these elevations in IRS-1 Ser^636/639^ phosphorylation did not reach the level of statistical significance. Cells pre-exposed for 16 hours to low T concentrations (20 ng/mL) had a significantly greater insulin-induced IRS-1 Ser^636/639^ phosphorylation compared to non-T exposed (control) cells (*p* = 0.042, figure 2B) following 30 minutes of exposure to 100 nM of insulin. Cells pre-exposed for 16 hours to high T concentrations (200 ng/ml) resulted in a non-significant increase in IRS-1 Ser^636/639^ compared to non-T exposed (control) cells (*p = *0.205, Figure 2B) following 30 minutes of exposure to 100 nM of insulin. Exposure to neither low-dose T nor high-dose T alone affected IRS-1 Ser^636/639^ phosphorylation.

#### Akt

**Figure 3:**
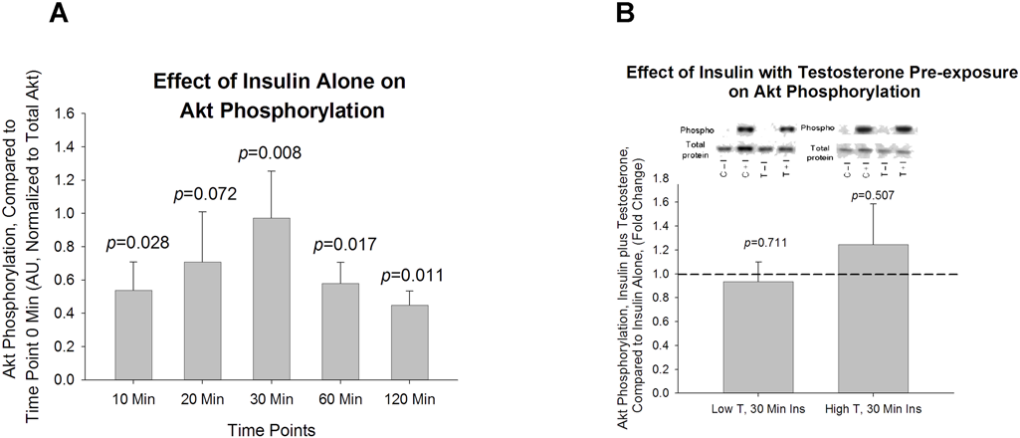
The effect of insulin in Akt Ser^473^ phosphorylation with or without pre-exposure of low and high dose testosterone. (A). The effect of insulin alone on Akt phosphorylation after insulin treatment for 10, 20, 30, 60, and 120 minutes; Insulin significantly increased Akt Ser^473^ phosphorylation at all time points except 20 minutes; (B). Cells were pre-exposed to low-dose (20ng/ml) or high-dose (200ng/ml) of testosterone for 16 hours before insulin treatment. No change in Akt Ser^473^ phosphorylation was observed between T treated and non-T treated cells. Representative western blots are shown above the bar graphs. The four experimental groups are: control cells without insulin (C−I), control cells plus insulin (C+I), testosterone exposed cells without insulin (T−I), and testosterone exposed cells plus insulin (T+I). The phosphorylated signal of Akt Ser^473^ is normalized to total Akt in the sample.

Exposure to 100 nM insulin resulted in increases in Akt phosphorylation at all time points: 10, 20, 30, 60, and 120 minutes (*p* = 0.028, *p* = 0.072, *p* = 0.007, *p* = 0.016, and *p* = 0.011, respectively, Figure 3A). Cells pre-exposed for 16 hours to both low (20 ng/mL) and high (200 ng/mL) T concentrations resulted in no significant changes in Akt phosphorylation compared to non-T exposed (control) cells (Figure 3B) following 30 minutes of exposure to 100 nM of insulin.

#### mTOR

**Figure 4:**
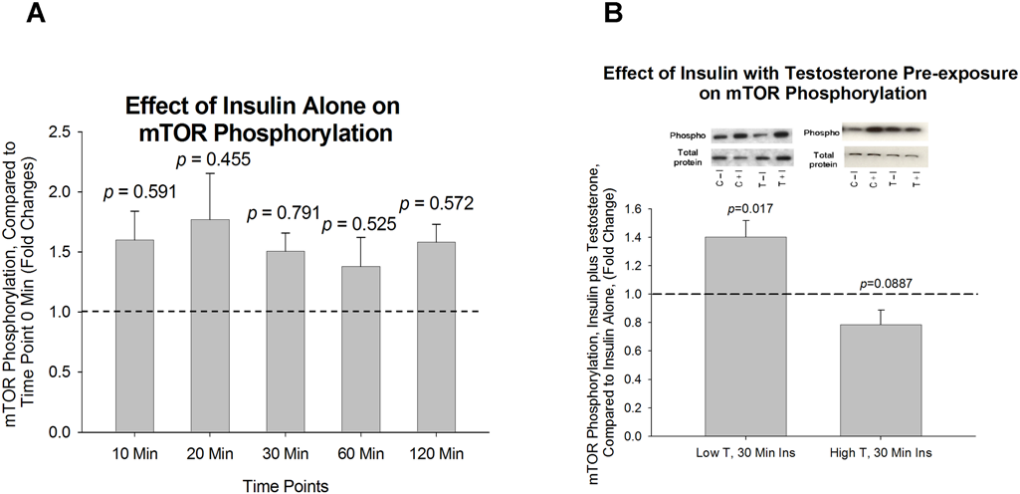
The effect of insulin in mTOR Ser^2448^ phosphorylation with or without pre-exposure of low and high dose testosterone. (A). The effect of insulin alone on mTOR Ser^2448^ phosphorylation after insulin treatment for 10, 20, 30, 60, and 120 minutes; Insulin had no effect on mTOR Ser^2448^ phosphorylation at all time points; (B). Cells were pre-exposed to low-dose (20ng/ml) or high-dose (200ng/ml) of testosterone for 16 hours before insulin treatment. Compared to non-testosterone treated cells, mTOR Ser^2448^ phosphorylation was significantly increased with low-dose testosterone treatment. Representative western blots are shown above the bar graphs. The four experimental groups are: control cells without insulin (C−I), control cells plus insulin (C+I), testosterone exposed cells without insulin (T−I), and testosterone exposed cells plus insulin (T+I). The phosphorylated signal of mTOR Ser^2448^ is normalized to total mTOR in the sample.

Exposure to 100 nM insulin at 10, 20, 30, 60, and 120 minutes did not change mTOR phosphorylation at any time point (figure 4A). Cells pre-exposed for 16 hours to low T concentrations (20 ng/mL) had a 40% increase in insulin-induced mTOR phosphorylation compared to non-T exposed (control) cells (*p = *0.017, Figure 4B) following 30 minutes of exposure to 100 nM of insulin. Cells pre-exposed for 16 hours to high T concentrations (200 ng/mL) resulted in a borderline-significant reduction in mTOR phosphorylation compared to non-T exposed (control) cells (*p = *0.088, Figure 4B) following 30 minutes of exposure to 100 nM of insulin.

#### S6K

**Figure 5:**
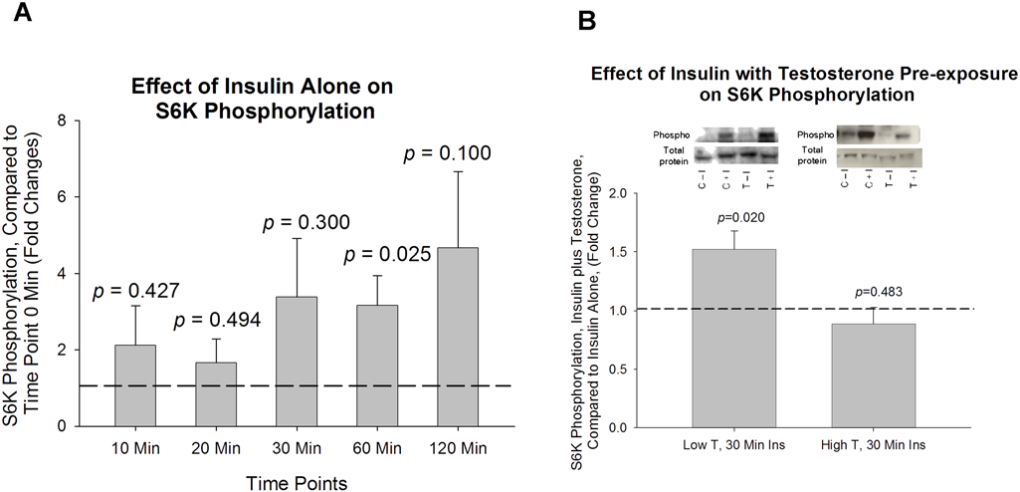
The effect of insulin in S6K Thr^389^ phosphorylation with or without pre-exposure of low and high dose testosterone. (A). The effect of insulin alone on S6K Thr^389^ phosphorylation after insulin treatment for 10, 20, 30, 60, and 120 minutes; Insulin significantly increased S6K Thr^389^ phosphorylation after 60 minutes; (B). Cells were pre-exposed to low-dose (20ng/ml) or high-dose (200ng/ml) of testosterone for 16 hours before insulin treatment. Compared to non-testosterone treated cells, S6K Thr^389^ phosphorylation was significantly increased with low-dose testosterone treatment. Representative western blots are shown above the bar graphs. The four experimental groups are: control cells without insulin (C−I), control cells plus insulin (C+I), testosterone exposed cells without insulin (T−I), and testosterone exposed cells plus insulin (T+I). The phosphorylated signal of S6K Thr^389^ is normalized to total S6K in the sample.

Exposure to 100 nM insulin resulted in a significant increase in S6K phosphorylation at time point 60 minute compared to that of baseline (*p* = 0.025, Figure 5A). The cells pre-exposed for 16 hours to low T concentrations (20 ng/mL) had a 52% increase in insulin stimulated S6K phosphorylation compared to non-T exposed (control) cells following 30 minutes of exposure to 100 nM of insulin (*p = *0.020, Figure 5B). When cells were pre-exposed to high T concentrations (200 ng/mL), there were no significant changes in S6K phosphorylation compared to that of non-T exposed (control) cells following 30 minutes of exposure to 100 nM of insulin (Figure 5B).

## Discussion

The current study reports a link between a hyperinsulinemic/hyperandrogenic environment and an elevation in serine phosphorylation of IRS-1, which has been associated with insulin resistance. More specifically, these results suggest there is a synergistic interaction between T and hyperinsulinemia in its effect beyond that of hyperinsulinemia alone. At both the low and high doses of T, IRS-1 demonstrated an ∼300% increase in insulin stimulated Ser^636/639^ phosphorylation. Regarding the Akt-mTOR-S6K pathway, low-dose testosterone exposure significantly increased both mTOR and S6K phosphorylation by insulin compared to those from control cells that were not pre-exposed to testosterone. There are other factors causing insulin resistance, known to be involved in elevated IRS-1 serine phosphorylation including tumor necrosis factor α (TNF-α), free fatty acids, cellular stress, amino acids, and insulin [16]. These molecules are thought to act on IRS-1 through a variety of serine/threonine kinases including mTOR and S6K [9], [12], [16], [17]. Therefore, low-dose testosterone seems to elevate insulin-induced IRS-1 serine phosphorylation similar to TNF-α, free fatty acids, amino acids, and insulin.

Of note, we did not observe any significant changes in insulin-associated mTOR/S6K phosphorylation when cells were pre-exposed to a higher dose of testosterone, which may be due to the desensitization of mTOR/S6K to insulin under current *in vitro* conditions. Other studies have suggested that mTOR's effect on IRS-1 Ser^636/639^ phosphorylation is mediated through TNF-α [17]. Our data demonstrating increased phosphorylation of mTOR and S6K paralleling that of IRS-1 Ser^636/639^ phosphorylation in response to a hyperandrogenic, hyperinsulinemic stimulus are consistent with these prior studies. Additionally, the observed increase in mTOR and S6K insulin stimulated phosphorylation following T exposure was not observed for Akt, which is consistent with the findings of Khamzina et al [9], which demonstrated elevated IRS-1 Ser^636/639^ resulted in suppression of Akt phosphorylation.

Increased IRS-1 Ser^636/639^ phosphorylation has previously been reported in liver and skeletal muscles of obese, insulin-resistant rats under insulin stimulation [9], which is similar to our observations where insulin alone significantly stimulated IRS-1 Ser^636/639^ phosphorylation at time point 120 minute (Figure 2A). The same study also suggested that suppressed Akt activity in liver and skeletal muscle of the obese animals is potentially due to the IRS-1 Ser^636/639^ phosphorylation secondary to insulin resistance, which uncouples PI3-kinase from the insulin receptor and results in reduced insulin signaling through Akt [9].

The results from the current study offer an opportunity to better understand the underlying mechanism of insulin resistance in PCOS patients. The association between hyperandrogenism and insulin resistance is well established in both animal models and humans. Multiple mechanisms have been suggested based on animal studies including reduced skeletal muscle capillary formation, muscle fiber isoform switching, reduced glycogen synthase activity, and impaired insulin-induced glucose transporter 4 (GLUT4) plasma membrane translocation [18]–[21]. However, much of the data regarding hyperandrogenic insulin resistance in humans has been observational [5], [6], [22], [23].

Studies of the mechanism of insulin resistance of PCOS have found evidence of altered serine phosphorylation in the insulin receptors of cultured skin fibroblasts from PCOS patients, which inhibited IR activation by tyrosine autophosphorylation in response to insulin stimulation [24], [25]. Of note, PCOS patients had significant elevations in both total and bioavailable T compared to the control subjects. A recent study using cultured skeletal muscle from 10 PCOS patients and matched control subjects reported a 35% significant increase in IRS-1 protein abundance in cultured skeletal muscle of PCOS patients, and the insulin stimulated IRS-1 associated PI3K activity was reduced in the PCOS patients [11]. In addition, insulin stimulated IRS-1 Ser^312^ was 55% higher in the PCOS patients as compared to controls, even after controlling for the elevation in IRS-1 protein abundance in these subjects [11]. The present study took the next logical step by examining the effects of both low-dose and high-dose testosterone exposure on insulin-induced phosphorylation of serine (inhibitory) residues on IRS-1 as well as some of its downstream targets. This finding from the current study is consistent with that of Courbold et al and demonstrates that increased insulin stimulated IRS-1 Ser phosphorylation occurs in cultured rat skeletal muscle after pre-exposure to T. Although there are at least 50 known potential serine/threonine phosphorylation sites on IRS-1, most studies relating IRS-1 serine phosphorylation to insulin resistance study one, or at most several, sites in response to a given stimulus [9], [11], [14], [16], [17], [26], [27]. Elevations in phosphorylation at both IRS-1 sites, Ser^312^ and Ser^636/639^, have been reported in other studies in association with insulin resistance [9], [17], [27].

The fact that we observed a similar trend to increase insulin-induced IRS-1 serine phosphorylation with both doses of T indicates that both the low- and high-dose T exposure enhanced insulin-induced IRS-1 serine phosphorylation. It should be noted that the low-dose T concentrations is at the upper limit of the physiological range, which demonstrated a significant IRS-1 serine phosphorylation and although the larger dose of T showed similar trend it did not reach the level of statistical significance. There may be multiple pathways and signaling proteins other than IRS-1 involved in this interaction between T and insulin. Previous studies suggested other pathways such as the mitogen activated protein kinase (MAPK) pathway and the stress activated protein kinase JNK, which are implicated in insulin induced IRS-1 serine phosphorylation and insulin resistance [28], [29].

Another intriguing finding of the present study is the observation that insulin stimulated IRS-1 serine phosphorylation in the absence of T, while concomitantly inhibiting the phosphorylation AKT beyond 30 minutes of insulin exposure (Figures 2A and 3A). This additional finding indicates that insulin can modulate its own signaling pathway. This finding is consistent with a recent report that indicated that continually high insulin levels impaired Akt phosphorylation and glucose transport in human myoblasts [30].

We chose an *in vitro* model to test our hypothesis. The *in vitro* model of the experimental design has limitations in that the model may not always accurately represent to *in vivo* biology. However, *in vitro* studies allow for the isolation of the effects of specific stimuli (in this case testosterone) on aberrant protein signaling (in this case insulin-stimulated IRS-1 Ser^636/639^ phosphorylation) from complexities of *in vivo* model. In addition, we chose to initially test our hypothesis in this primary rat skeletal muscle myotubes, but future investigations are warranted to determine whether T exposure has similar effects on insulin-stimulated IRS-1 Ser^636/639^ phosphorylation in primary human skeletal muscle myotubes as well as *in vivo* in humans.

In conclusion, we report a specific link between a hyperinsulinemic, hyperandrogenic environment and an elevation of IRS-1 serine phosphorylation of IRS-1, which has been associated with the development of insulin resistance. Specifically, our results suggest that there is a synergistic interaction between T and insulin in this effect beyond that of insulin alone. Additionally, we suggest future studies utilizing primary skeletal muscle culture exposed to T in the presence of a hyperinsulinemic environment may serve as models for PCOS-related insulin resistance. These results agree with the recent findings of elevated serine phosphorylation of IRS-1 in cultured skeletal muscle of PCOS patients [11].

## Materials and Methods

### Skeletal Muscle Culture

The study protocol was approved by the Institutional Animal Care and Use Committee of Mayo Clinic and Foundation. Skeletal muscle was obtained from the soleus and EDL muscle of an adult female Sprague-Dawley rat from which myoblasts were isolated and cultured according to established protocols [31]–[33]. Specifically, muscle biopsy samples were collected in ice-cold phosphate-buffered saline (PBS) supplemented with 1% PeSt (100 units/ml penicillin/100 μg/ml streptomycin). Fat and connective tissue were dissected from the specimen in a sterile tissue culture hood. The muscle was placed into a filter-sterilized collagenase solution (0.2% type I collagenase (Sigma) in Dulbecco's modified Eagles medium (DMEM, Sigma) and incubated for 1.5 h at 37°C in an oscillating water bath. Following digestion, the muscle cells were transferred to a 100mm petri dish (Falcon) pre-rinsed with DMEM containing horse serum (HS). A wide-bore pipet (Falcon) was then used to gently separate muscle fibers while the mass of muscle cells were then transferred to a fresh identically-prepared petri dish and the separation process repeated. The supernatant from each separation process was transferred to a non-coated 100mm petri dish and incubated for 1 h in a tissue culture incubator at 37°C and 5% CO_2_ to promote adherence of non-myogenic cells. The non-adherent cells from each non-coated dish were then transferred to a 24-well tissue culture plate (Falcon) precoated with 10% Matrigel and allowed to settle and attach for 3 min prior to the placement of 0.5 ml plating media (DMEM, 10% HS, 0.5% chick embryo extract (CEE), 1% PeSt) per well. After 3 days, the media was changed to proliferation media containing 20% fetal bovine serum (FBS), 10% HS, 0.5% CEE, and 1% PeSt. The cells, when reached ∼80% confluence, were then subcultured according to standard techniques and transferred to non-coated 75-cm^2^ flasks (Falcon) for continued culture.

Myoblasts were differentiated into multi-nucleated myotubes after 4 days in low-serum media containing DMEM, 2% FBS, and 1% PeSt. All protein experiments were performed using myotubes.

### Immunohistochemical (IHC) Staining

Myoblasts, upon reaching 80% confluence, were trypsinized and mounted on microscope slides for homogeneity estimation with IHC staining using the myoblast-specific myosin heavy chain-1 (MHC-1) antibody (Sigma, M8421) Briefly, following trypsinization the cells were suspended in 1% bovine serum albumin (BSA)/PBS. The cell concentration was adjusted to 1×10^5^ cells/ml by serial dilution. Two-hundred microliters of the cell suspension was loaded into the cytofunnel to provide a final concentration of 2×10^4^ cells per slide. The cells were spun at 600 rpm for 5 min to mount them on the slide. The slides were dried for 2 hours prior to antibody staining. Following drying, each slide was washed with PBS times for 1–2 min, followed by acetone fixation for 1 min. The slides were dried for 10 min, followed by an additional 1 min PBS wash. The slides were then incubated for 10 min at room temperature (RT) in 100 μl of Dako Cytomation Protein Block (DPBB) (DakoUSA x0909) with 1% saponin (Sap) (Sigma S-7900 100X stock). Slides were exposed to MHC-1 antibody (Ab) diluted 1:500 in DPBB-Sap for 1 hr at RT. Negative control slides were incubated in DPBB-Sap for 1 hr at RT. Slides were then washed 3 times in PBS for 5 min. Secondary Ab (Molecular Probes Alexa Flour 488) was applied for 30 min. Following PBS wash, slides were counterstained with a Hoescht counterstain (Sigma B2883). Slides were then mounted with an anti-fade agent (Molecular Probes P7481) and inspected using a Zeiss LSM 510 confocal laser scanning microscope (Carl Zeiss, Inc., Germany) set for excitation at 488 nm utilizing an argon/krypton laser. Negative control slides were used to calibrate the microscope prior to image analysis of the MHC-1 slides.

### Western Blot Analysis

Sixteen hours prior to insulin exposure, differentiated myotube cells were adapted to serum-free media containing 1% BSA without testosterone (control, C) or with testosterone (T) at two different concentrations, 20 ng/mL and 200 ng/mL. Then C and T pre-exposed cells were treated with or without 100 nM insulin (I) resulting in 6 experimental groups: C−I, C+I low T−I, low T+I, high T−I and high T+I. The T concentrations were empirically chosen. In brief, we used the upper physiologic limit for T concentration for the low-dose T and multiplied it by a factor of 10 base on our understanding that metabolic processes occur at much faster rates in rats. We then performed preliminary Western blots to determine if we could see a difference in protein phosphorylation. Based on our preliminary results we designed the experiment to compare the 2 concentrations. The 100 nM insulin concentration was chosen, because it represents a standard *in vitro* insulin concentration for stimulating glucose uptake [34]. Cells and total proteins from the cells were collected using cell lysis buffer supplemented with protease inhibitor cocktail (Cell Signaling #9803, Roche complete mini #1836153) after 30 minutes, 1 hour, and 2 hours of insulin stimulation for the low-dose T pre-exposed cells, and after 10, 20 and 30 min of insulin stimulation for the high-dose T treated cells . For both the low-dose and high-dose T exposed cells, the 30 min data is presented based on preliminary experiments which suggested that at this time point the phosphorylation status of IRS1 Ser^636/639^ and S6k plateaued, Akt reached its peak phosphorylation, and mTOR phosphorylation remained unchanged. Total proteins were separated by gel electrophoresis using the Invitrogen Mini Cell^©^ system and NuPage 4–12% Tris-Bis gels. Proteins were then transferred to PVDF membranes, and western blot analysis was performed using antibodies against total and phosphorylated Akt Ser^473^ (Cell Signaling #9272, #9271), mTOR Ser^2448^ (Cell Signaling #2972, #2971), S6 kinase Thr^389^ (Cell Signaling #9202, #9205), and IRS-1Ser^636/639^ (Cell Signaling #2382, #2388). Signal intensities of phosphorylated and total proteins were quantified and analyzed using Kodak image station 1000 and the accompanying software packages. Phosphorylated signals were normalized to total protein for each measurement. The net effect of insulin on protein phosphorylation was determined using the ratio of the insulin-treated phosphorylation signal to the non-insulin treated phosphorylation signal for both control cells and T exposed cells. The experiment was replicated six times for each condition.

### Statistics

Data are presented as the mean±SE. Statistical significances were calculated using unpaired t-tests.
